# Autism and anorexia nervosa: Longitudinal prediction of eating disorder outcomes

**DOI:** 10.3389/fpsyt.2022.985867

**Published:** 2022-09-21

**Authors:** Jenni Leppanen, Felicity Sedgewick, Daniel Halls, Kate Tchanturia

**Affiliations:** ^1^Department of Neuroimaging, Institute of Psychology, Psychiatry and Neuroscience, King’s College London, London, United Kingdom; ^2^School of Education, University of Bristol, Bristol, United Kingdom; ^3^Department of Psychological Medicine, Institute of Psychology, Psychiatry and Neuroscience, King’s College London, London, United Kingdom; ^4^South London and Maudsley NHS Foundation Trust National Eating Disorder Service, London, United Kingdom; ^5^Department of Psychology, Illia State University, Tbilisi, Georgia; ^6^Psychological Set Research and Correction Center, Tbilisi State Medical University, Tbilisi, Georgia

**Keywords:** anorexia nervosa, autism, autistic traits, eating disorder symptoms, depression, anxiety, ADOS, AQ10

## Abstract

**Background:**

Recently, elevated levels of autistic features and autism diagnoses have been reported among people with anorexia nervosa (AN). In clinical settings high levels of autistic features have been linked to more complex, highly comorbid illness presentation and poorer treatment outcome. This study aimed to examine whether autistic features predict AN symptom profile in long term.

**Methods:**

Altogether 118 women with lived experience of AN completed two autism assessments at time 1, the Autism Diagnostic Observation Schedule (ADOS) and the short version of the Autism Quotient (AQ10). Measures assessing AN symptom profile, including eating disorders symptoms, anxiety, depression, OCD symptoms, and Body Mass Index (BMI), were also recorded. The symptom profile measures were administered again 6 months and 2 years later. We conducted two analyses to examine the extent to which the ADOS and AQ10 scores predicted broad AN symptom profile at each three time points.

**Results:**

Overall, high levels of autistic features were consistently associated with worse psychological symptoms, but not BMI, across all time points. Both the analysis using baseline ADOS scores and self-reported AQ10 scores showed similar pattern.

**Conclusion:**

The present findings consolidate previously reported associations between autistic features and worse psychological outcome among people with AN. The findings also suggest that self-report measures may be sufficient for assessing the impact of autistic features on illness outcome among people with AN. Importantly, the study highlights the need for development and further investigation of neurodiversity accommodations in the treatment of AN.

## Introduction

Anorexia nervosa (AN) is a complex psychiatric disorder, characterized by intense fear of gaining weight and subsequent malnutrition (1, 2). It has been estimated that between 45 and 97% of people with AN have at least one comorbid psychiatric disorder (3). The most prevalent comorbid disorders are depression, anxiety disorders and obsessive-compulsive disorder (OCD) (4–6). Although adolescents AN patients with shorter duration of illness have been found to have lower rate of comorbid psychiatric disorders than adults, it has been estimated that up to 60% of adolescents with AN also have mood disorders, while up to 16% also have comorbid anxiety disorder (7, 8). Importantly, highly comorbid presentation of AN has been linked to greater severity and longer duration of illness (6), which may go some way to explain the age-related differences in the prevalence of comorbid diagnoses. Together these findings suggest that AN frequently has complex psychopathology and symptom profile.

Previous research has documented that in addition to high rates of comorbid psychiatric disorders, somewhere between 4 and 33% of people with AN also meet criteria for possible autism^1^ based on self-report while 2–53% met diagnostic cut-off for autism based on clinician assessment (9–12). Autism is a neurodevelopmental condition present from early childhood, but it is often missed among girls and women resulting in late diagnosis during adolescence or adulthood (13–15). Autism is typically associated with differences in cognitive style, including greater detail focus and difficulties making top-down predictions about the world which are in turn linked to intolerance of uncertainty, cognitive rigidity and greater desire for sameness (16). In addition to cognitive differences, autism is often associated with alterations in social communication and interaction (17, 18). Autistic people typically communicate and experience the world in a different way compared to neurotypical people (18, 19). This results in a gap in communication expectations, also known as the double empathy problem, such that both autistic and neurotypical people struggle to relate to and empathize with one another (17–19). Similar differences in cognitive style and social functioning have been documented in AN, particularly among those with long duration of illness (20).

Autistic social and cognitive styles clashing with the neurotypical world has been proposed to underlie high levels of mental health problems in autism, which are further compounded by other factors including stigma, trauma, and prejudice (16, 19, 21). In the same vein, in the field of eating disorders (ED), cross-sectional studies have documented that those who report more autistic features present more severe ED psychopathology and complex illness profiles, with more comorbid anxiety, depression, and OCD symptoms (22, 23). Furthermore, previous longitudinal, naturalistic studies of AN patients admitted to inpatient ED services found that those who self-reported higher levels of autistic features also reported more ED symptoms, depression and anxiety, and poorer social functioning at both admission and discharge (24–26). However, notably the average duration inpatient treatment was 16 weeks making it difficult to draw long-term conclusions from these findings. Additionally, other recent studies have not reported significant association between autistic features and ED symptoms or differences in illness outcome between AN patients with and without high levels of autistic features (27, 28). Therefore, it is of interest to examine whether and to what extent autistic features can predict complex illness presentation in AN in long term.

The present study aims to build on the previous literature by examining whether autistic features measured at baseline can predict ED symptom profile among those with AN at later time points. We additionally aimed to explore whether a short, self-report screening measure could be used to predict ED symptom profile over time in a similar fashion as a gold standard diagnostic tool. Based on the previous work outlined above, we hypothesized that autistic features would consistently predict worse illness presentation over time. Additionally, we hypothesize that this relationship would not change significantly over time as autistic features are meant reflect a neurotype and remain stable over time.

## Materials and methods

### Participants

Altogether 142 people with lived experience of AN expressed interest in taking part in a large neuroimaging study conducted between June 2017 and February 2019, which examined neural underpinnings of AN (29–32). Participants were included if they identified as female, had a current or past diagnosis of AN, had not experienced significant head injury, had no learning disabilities, and were able to undergo magnetic resonance imaging (MRI). Twenty-one women declined taking part or dropped out of the study after initial screening and three women were excluded for MRI incompatibility. The present study used data collected during the neuroimaging study and included 118 participants who were followed-up across three time points. All participants were female aged 12–27 with lived experience of AN ranging from acute illness to fully recovered. To establish illness status, participants were asked whether they considered themselves recovered, experiences ED symptoms, and used ED-services. Those who at the first assessment considered themselves recovered, were no longer reported experiencing symptoms and did not use or need ED services were classed fully recovered. Other participants were considered to be in various stages of acute illness. Most participants were white Europeans while 7.3% reported mixed heritage and 2.5% were of Asian heritage. The average duration of illness was 3.7 years (standard deviation = 2.8). All participants were recruited through flyers posted in local NHS eating disorder services in London and online through BEAT eating disorders charity. Participants provided written, informed consent prior to taking part in the study. The study procedures were all completed in accordance with the latest version of the Declaration of Helsinki (2013) and the study was approved by The London-Surrey National Research Ethics Committee (17/LO/2071 and 19/SC/0367).

### Assessments

Autistic features were assessed using the Autism Diagnostic Observation Schedule –Second Edition, Module 4 [ADOS-2, (33, 34)] and the short version of the Autism Quotient self-report questionnaire [AQ10, (35)]. The ADOS-2 is a gold standard observational module used to in the diagnosis of autism based on the criteria in the 4th edition of the Diagnostic and Statistical Manual of Mental Disorders. In the present study, we used the revised algorithm was used to calculate a total score of autism features, which was a sum of the social affect and restrictive and repetitive behaviors subscales (34). A score above the cut-off of 8 is considered to indicate autism diagnosis. The AQ10, on the other hand, is a brief self-report screening measure for autistic features. AQ10 total score above the cut-off of 6 is considered to indicate possible autism.

Eating disorders symptom profile was assessed by collecting participants’ height and weight to calculate BMI and by using the following self-report questionnaires: The Eating Disorder Examination Questionnaire [EDEQ, (36)], Hospital Anxiety and Depression Scale [HADS, GL Assessments ref. 629398237, (37)], Obsessive-Compulsive Inventory [OCI, (38)], and Work and Social Adjustment Scale [WSAS, (39)].

### Procedures

At the first time point, Time 1, participants completed the AQ10 and ED symptom profile self-report questionnaires. A subset of the participants also agreed to complete the ADOS-2 assessment (*N* = 73, 70% of the sample) at Time 1. ADOS-2 was delivered by a trained examiner and took approximately 45–60 min to complete. Participants’ height and weight were measured after the ADOS-2 assessment and were used to calculate body mass index (BMI). The Time 1 assessments were a part of a larger study conducted between June 2017 and February 2019, which examined neural underpinnings of AN (29–32).

At Time 2, 6 months after the Time 1 assessments, participants were asked to complete the ED symptom profile self-report questionnaires. Participants were also asked to report their current height and weight for BMI calculation. At Time 3, 2 years after the initial Time 1 assessments, participants were asked to again complete the ED symptom profile questionnaires and report their current height and weight.

### Data analysis

All data analysis was conducted in R (40). We first conducted a principal component analysis to reduce dimensionality of the outcome dataset. This was done by applying the function *prcomp* to the measures used to assess ED symptom profile, which included BMI, EDEQ total score, HADS anxiety score, HADS depression score, OCI total score, and WSAS score. We used the elbow method to determine the number of components that should be taken forward to further analysis. The retained principal components were then entered as outcome variables to two multivariate linear mixed effects models (LMERs) with the following predictors: Model (1) Time 1 ADOS-2 total score and time point, Model (2) Time 1 AQ10 total score and time point. The multivariate LMERs were conducted using the *lme4* package (41). Threshold for statistical significance was set at *p* < 0.025 after adjusting for the two LMER tests. The data and code used in the analyses are available at https://osf.io/tqysn/?view_only=69a0f2183df14f5e82f4b728fba50f34.

## Results

### Sample characteristics

Clinical and demographic sample characteristics at each time point are reported in Table 1. Of those who completed the autism assessments, 30 (41%) participants scored above the cut-off on the ADOS-2 while 35 (33%) scored above the cut-off on the AQ10. Nine (12%) participants scored above the cut-off on both measures. Thirteen participants did not complete all initial Time 1 questionnaires but did complete the follow-up questionnaires at Time 2 and 3.

**TABLE 1:** Clinical and demographic sample characteristics.

	Time 1 (*N* = 105)	Time 2 (*N* = 82)	Time 3 (*N* = 86)
Age	18.9 (3.26)	19.3 (2.97)	21.6 (3.50)
BMI	18.4 (2.80)	18.1 (2.74)	18.6 (2.63)
EDEQ total	3.1 (1.60)	2.8 (1.70)	2.5 (1.6)
HADS anxiety	10.4 (3.69)	11.0 (5.07)	10.9 (4.76)
HADS depression	7.6 (4.47)	7.0 (5.39)	6.6 (4.49)
OCI score	20.3 (14.08)	23.7 (16.68)	20.5 (15.01)
WSAS score	17.7 (10.61)	16.2 (12.81)	14.5 (11.51)

BMI, body mass index; EDEQ, Eating Disorder Examination Questionnaire; HADS, Hospital Anxiety and Depression Scale; OCI, Obsessive-Compulsive Inventory; WSAS, Work and Social Adjustment Scale.

### Longitudinal analysis

All variables that were entered in the principal component analysis were significantly correlated at each time point except for the following correlations at Time 1 BMI and EDEQ Total score, BMI and HADS anxiety, BMI and WSAS score, and BMI and OCI Score (Supplementary Table 1). Additionally, BMI was not significantly correlated with OCI Scores at Time 3. The elbow method indicated that the first two principal components, which explained 71.4% of the variance in the data, should be retained (Supplementary Figure 1 and Supplementary Table 2). The first principal component, PC1, was negatively associated with the WSAS score, EDEQ total score, HADS depression, HADS anxiety, and OCI score (Supplementary Table 2). The second principal component, PC2, was primarily positively associated with BMI.

**FIGURE 1:**
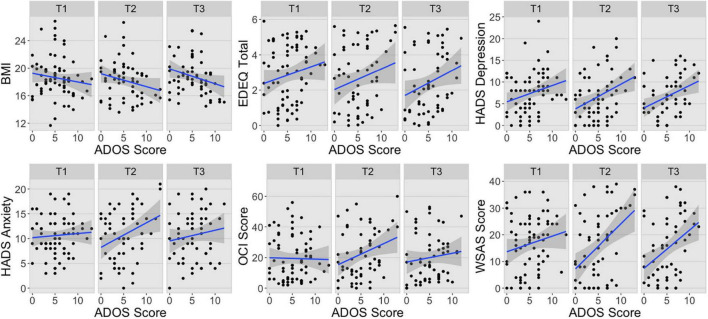
Associations between ADOS scores and ED symptom profile measures at each time point. BMI, body mass index; EDEQ, Eating Disorder Examination Questionnaire; HADS, Hospital Anxiety and Depression Scale; OCI, Obsessive-Compulsive Inventory; WSAS, Work and Social Adjustment Scale; T1, Time 1; T2, Time 2; T3, Time 3.

There was a significant interaction between the principal components and ADOS-2 scores in the first multivariate LMER suggesting that ADOS-2 total score was differently associated with the two principal components (*F*(1,298) = 12.79, *p* < 0.001; Table 2 and Supplementary Figure 2). Indeed, there was a stronger negative association between ADOS-2 scores and PC1 (*b* = −0.19, 95% CI [−0.26, −0.11]) than between ADOS-2 scores and PC2 (*b* = −0.04, 95% CI [−0.11, 0.04]) (*t*(267) = −3.58, *d* = −0.11 95% CI [−0.18, −0.05], *p* < 0.001). These results together with visual inspection of the associations between ADOS scores and the ED symptom profile measures presented in Figure 1, show higher level of autistic features generally predicted worse presentation across all three time points, with the exception of anxiety and OCD symptoms. The first multivariate LMER also revealed significant main effects of principal component and ADOS-2 total score, but these were not interpreted or explored further due to the presence of a significant interaction. There were no other significant main effects or interactions.

**TABLE 2:** Association between Time 1 ADOS total and AQ10 scores, and ED symptom profile across time points.

Measure	Time point			F(DF)-statistic, *p*-value	
	1	2	3	ADOS (*N* = 65)	AQ10 (*N* = 105)
ADOS total	5.27 (3.07)	–	–	PC: *F*(1,298) = 11.51, *p* = 0.001	PC: *F*(1,403) = 52.98, *p* < 0.001
AQ10	3.83 (2.24)	–	–	ADOS: *F*(1,80) = 11.38, *p* = 0.001	AQ10: *F*(1,109) = 11.06, *p* = 0.001
PC1	−0.12 (1.58)	−0.08 (2.17)	0.23 (1.81)	Time point: *F*(2,328) = 0.79, *p* = 0.454	Time point: *F*(2,434) = 1.04, *p* = 0.354
PC2	−0.06 (0.96)	0.00 (0.94)	0.08 (0.92)	PC x ADOS: *F*(1,298) = 12.79, *p* < 0.001 PC x Time point: *F*(2,280) = 1.38, *p* = 0.252 ADOS x Time point: *F*(2,329) = 0.54, *p* = 0.583 PC x ADOS x Time point: *F*(2,298) = 1.75, *p* = 0.175	PC x AQ10: *F*(1,403) = 67.55, *p* < 0.001 PC x Time point: *F*(2,403) = 2.28, *p* = 0.104 AQ10 x Time point: *F*(2,438) = 1.05, *p* = 0.349 PC x AQ10 x Time point: *F*(2,403) = 2.94, *p* = 0.054

ADOS, Autism Diagnostic Observation Schedule; PC, principal component; DF, degrees of freedom.

**FIGURE 2:**
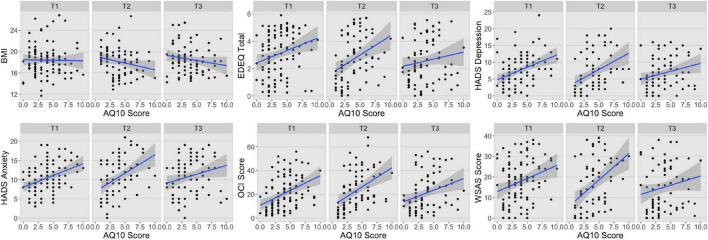
Associations between AQ10 scores and ED symptom profile measures at each time point. BMI, body mass index; EDEQ, Eating Disorder Examination Questionnaire; HADS, Hospital Anxiety and Depression Scale; OCI, Obsessive-Compulsive Inventory; WSAS, Work and Social Adjustment Scale; T1, Time 1; T2, Time 2; T3, Time 3.

The second multivariate LMER similarly showed a significant interaction between principal component and Time 1 AQ10 scores (Table 2 and Supplementary Figure 2). As above, the AQ10 scores were differently associated with PC1 and PC2 (*t*(400) = −8.22, *d* = −0.33 95% CI −0.41, −0.25], *p* < 0.001), such that there was a negative association between AQ10 scores and PC1 (*b* = −0.32, 95% CI [−0.41, −0.23]) and only a slight positive association between AQ10 scores and PC2 across the time points (*b* = 0.06, 95% CI [−0.04, 0.14]). Together with a visual inspection of the associations between Time 1 AQ10 scores and the ED symptom profile measures presented in Figure 2, the results suggest that higher level of autistic features predicted more psychological symptoms across time points but was not associated with BMI. As above there were also significant main effects of principal component and Time 1 AQ10 scores, but these were not interpreted or explored further due to the presence of a significant interaction. There were no other significant main effects or interactions.

## Discussion

The present study investigated whether baseline autistic features can predict ED symptom profile at a later date in a diverse sample of young women with lived experience of AN. The findings revealed that high baseline autistic features were consistently associated with worse psychological symptoms even 2 years later. Interestingly, both the brief self-report and the diagnostic tool used to assess autistic features similarly predicted psychological ED symptoms, but not BMI, which is usually one of the key outcome measures in AN research and treatment. Further visual examination of each symptom measure showed some differences between the two autism assessment tools.

The present findings lend further credence to previous work documenting that people with AN who report high levels of autistic features have more severe psychological presentation (25, 26, 42, 43). Furthermore, the present findings add to the literature by highlighting that high levels of autistic features predict poorer psychological outcome in long term. Although 15.3% of participants in the present study considered themselves recovered and were no longer in treatment for AN, this is in line with qualitative findings that many autistic people with EDs report that currently available treatments do not adequately meet their needs forming a further barrier to recovery (44, 45). Additionally, many clinicians working in ED services lack the experience and confidence needed to identify autism and effectively meet the needs of autistic ED patients, despite acknowledging that treatment adaptations are needed (46). Together, these findings highlight the importance of additional training for clinicians and neurodiversity-focused treatment adaptations to improve ED outcomes among autistic people and those with high levels of autistic features.

The present findings show that high levels of autistic features have a strong association with complex psychological ED symptom profile, but not BMI. Although it is important to note that the present study included adolescents as young as 12 and that BMI may not be a very informative measure with such as young population, previous studies have documented similar lack of association between autistic features and BMI among adult AN patients (23, 26). A recent examination of clinical audit data also showed that adult AN patients reporting high levels of autistic features had significantly higher BMI upon admission to inpatient care than those reporting lower level of autistic features (42). Moreover, sole focus on BMI as the indicator of recovery has been highlighted as less helpful for autistic people with AN, who report that the psychological elements of recovery are often more difficult to manage (47). Along the same lines, qualitative work exploring the experiences of autistic people with EDs has found that body image difficulties and drive for thinness are less relevant for this group of patients (44, 45). This may further help to explain why autism symptomatology in this sample predicts psychological ED symptoms 2 years later, but not BMI and physical recovery. These findings highlight a need for a shift in thinking about the illness presentation among autistic ED patients and those with high levels of autistic features with greater focus on psychological wellbeing and outcomes.

As autism is being increasingly recognized in ED treatment, there has been a great deal of interest in developing and investigating methods to identity those ED patients who might require neurodiversity-focused treatment adaptations (48–50). Although, both the AQ10 and the ADOS-2 appeared to have sufficient predictive power in the present study, these methods have also received some criticism. Self-report screening measures such as the AQ10, are convenient, but they cannot be used to diagnose autism and some have shown poor performance in accurately identifying autistic people (51). Although clinician administered tools, such as the ADOS-2, are used in the diagnostic process, they are expensive and require extensive training. Additionally, previous work has criticized the use of measures that solely focus on current features, tendencies, and behaviors, suggesting that they may not be beneficial in detecting autistic features in people with acute ED due to lack of exploration of the person’s developmental history (11, 52). This is particularly relevant in the field of EDs as it has been suggested that malnutrition in AN and long duration of illness can produce in autism-like behaviors (53, 54). Therefore, it may be of interest to investigate whether including retrospective questions exploring developmental history would help improve the robustness of self-report measures to identity autistic ED patients and those with high levels of autistic features.

Thus far, few treatment adaptations have been introduced to better serve autistic ED patients (55–57). A recent review synthesized findings from nine studies exploring various ways to adapt interventions based on cognitive remediation therapy (CRT), emotion skills training and cognitive behavioral therapy (CBT) to better suit the needs of autistic ED patients (58). The authors also reported that autistic ED patients, or those with high levels of autistic features, may be less likely to benefit from standard interventions, which frequently focus on altering the autistic social and cognitive processing styles. Additionally, across the studies it was apparent that autistic ED patients or those with high levels of autistic features are unlikely to benefit from group interventions and may favor individual therapy. Only two of the studies reviewed explored the impact of proposed treatment adaptations: one presented data from a one-time sensory wellbeing workshop (56) and the other presented a case example of adaptations to the Maudsley Anorexia Nervosa Treatment for Adults [MANTRA; (57)]. Although both studies resulted in some improvements among AN patients with high levels of autistic features, there is relative paucity of research exploring the impact of neurodiversity accommodations on ED treatment outcomes and more work is needed.

### Limitations

Although the findings from the present study are important, there are also some limitations. Firstly, only 70% of the participants who completed the self-report questionnaires at Time 1 also agreed to undergo ADOS-2 assessment. This reduced the power of the LMER involving ADOS-2 scores when compared to the analysis involving AQ10 scores. Additionally, the sample consisted of only young women, and it is therefore, difficult to ascertain whether these findings extend to other sexes and genders and older age groups. Finally, the outcome measures were self-reported and participants’ height and weight could only be measured by the researchers at Time 1 because subsequent assessments were completed online. This may have impacted the results, particularly regarding BMI.

## Conclusion

High levels of autistic features, assessed using a self-report screening questionnaire and a clinician administer tool, were associated with more severe ED psychopathology and psychiatric comorbidities in long term. Interestingly, autistic features were not associated with BMI, which is supported by previous work reporting that body image and drive for thinness may be less relevant for autistic people with EDs. Finally, the present study highlights the need for the development and investigation of neurodiversity focused treatment adaptations.

## Data availability statement

The datasets presented in this study can be found in online repositories. The names of the repository/repositories and accession number(s) can be found below: https://osf.io/tqysn/?view_only=69a0f2183df14f5e82f4b728fba50f34.

## Ethics statement

The studies involving human participants were reviewed and approved by London-Surrey National Research Ethics Committee (17/LO/2071 and 19/SC/0367). The patients/participants provided their written informed consent to participate in this study.

## Author contributions

JL, FS, and DH collected the data used in the manuscript, interpreted the results, and made major contributions to writing the manuscript. KT and JL obtained funding for the study. KT additionally interpreted the results regarding clinical implications and made major contributions to the writeup. All authors read and approved the final manuscript.

## Footnotes

1 To respect the preferences of the autism community, we use the term “autism” and identity-first language when referring to autistic people throughout the manuscript (59, 60).
