# Supplementary material for: Autism and anorexia nervosa: Longitudinal prediction of eating disorder outcomes
Source: Front Psychiatry. 2022 Sep 21;13:985867. doi: 10.3389/fpsyt.2022.985867 (PMC9533087; doi:10.3389/fpsyt.2022.985867)
Supplement: Supplementary file 1 [file Table_1.DOCX]

Supplementary Table 1. Correlations between ED symptom profile variables at each time point

| Time point |  | BMI | EDEQ Total | HADS Anxiety | HADS Depression | WSAS Score | OCI Score |
| --- | --- | --- | --- | --- | --- | --- | --- |
| T1 | BMI | - | r = -0.13, p = 0.403 | r = -0.16, p = 0.337 | r = -0.29, p = 0.013 | r = -0.29, p = 0.199 | r = -0.10, p = 0.403 |
|  | EDEQ Total |  | - | r = 0.40, p < 0.001 | r = 0.53, p < 0.001 | r = 0.56, p < 0.001 | r = 0.36, p < 0.001 |
|  | HADS Anxiety |  |  | - | r = 0.58, p < 0.001 | r = 0.39, p < 0.001 | r = 0.45, p < 0.001 |
|  | HADS Depression |  |  |  | - | r = 0.55, p < 0.001 | r = 0.50, p < 0.001 |
|  | WSAS Score |  |  |  |  | - | r = 0.34, p = 0.003 |
|  | OCI Score |  |  |  |  |  | - |
| T2 |  | BMI | EDEQ Total | HADS Anxiety | HADS Depression | WSAS Score | OCI Score |
|  | BMI | - | r = -0.48, p < 0.001 | r = -0.28, p = 0.011 | r = -0.41, p < 0.001 | r = -0.54, p < 0.001 | r = -0.32, p = 0.007 |
|  | EDEQ Total |  | - | r = 0.67, p < 0.001 | r = 0.61, p < 0.001 | r = 0.71, p < 0.001 | r = 0.67, p < 0.001 |
|  | HADS Anxiety |  |  | - | r = 0.61, p < 0.001 | r = 0.70, p < 0.001 | r = 0.75, p < 0.001 |
|  | HADS Depression |  |  |  | - | r = 0.84, p < 0.001 | r = 0.53, p < 0.001 |
|  | WSAS Score |  |  |  |  | - | r = 0.58, p < 0.001 |
|  | OCI Score |  |  |  |  |  | - |
| T3 |  | BMI | EDEQ Total | HADS Anxiety | HADS Depression | WSAS Score | OCI Score |
|  | BMI | - | r = - 0.30, p = 0.013 | r = - 0.25, p = 0.041 | r = -0.35, p = 0.004 | r = -0.38, p = 0.002 | r = -0.16, p = 0.134 |
|  | EDEQ Total |  | - | r = 0.51, p < 0.001 | r = 0.58, p < 0.001 | r = 0.70, p < 0.001 | r = 0.50, p < 0.001 |
|  | HADS Anxiety |  |  | - | r = 0.51, p < 0.001 | r = 0.55, p < 0.001 | r = 0.50, p < 0.001 |
|  | HADS Depression |  |  |  | - | r = 0.74, p < 0.001 | r = 0.37, p = 0.003 |
|  | WSAS Score |  |  |  |  | - | r = 0.39, p = 0.001 |
|  | OCI Score |  |  |  |  |  | - |

Pearson correlation coefficients and corresponding p-values are presented in the upper triangle. The p-values were corrected for multiple comparisons using Holm's method.
