# Supplementary material for: Autism and anorexia nervosa: Longitudinal prediction of eating disorder outcomes
Source: Front Psychiatry. 2022 Sep 21;13:985867. doi: 10.3389/fpsyt.2022.985867 (PMC9533087; doi:10.3389/fpsyt.2022.985867)
Supplement: Supplementary file 2 [file Table_2.DOCX]

Supplementary Table 2. Results from the principal component analysis

| ED measure | PC1 | PC2 | PC3 | PC4 | PC5 | PC6 |
| --- | --- | --- | --- | --- | --- | --- |
| BMI | 0.26 | 0.86 | -0.44 | 0.03 | -0.07 | 0.03 |
| EDEQ total | -0.44 | 0.05 | -0.29 | -0.66 | 0.42 | 0.32 |
| HADS Anxiety | -0.42 | 0.30 | 0.28 | 0.57 | 0.58 | -0.04 |
| HADS depression | -0.45 | -0.07 | -0.32 | 0.35 | -0.53 | -0.53 |
| WSAS total | -0.46 | -0.10 | -0.42 | 0.05 | -0.08 | 0.77 |
| OCI total | -0.38 | 0.40 | 0.61 | -0.33 | -0.44 | 0.14 |
| Importance of components |  |  |  |  |  |  |
| SD | 1.84 | 0.94 | 0.79 | 0.68 | 0.61 | 0.51 |
| Proportion of variance | 0.57 | 0.15 | 0.10 | 0.08 | 0.06 | 0.04 |
| Cumulative variance | 0.57 | 0.71 | 0.82 | 0.89 | 0.96 | 1.00 |
