# Supplementary figures and images for: Autism and anorexia nervosa: Longitudinal prediction of eating disorder outcomes
Source: Front Psychiatry. 2022 Sep 21;13:985867. doi: 10.3389/fpsyt.2022.985867 (PMC9533087; doi:10.3389/fpsyt.2022.985867)

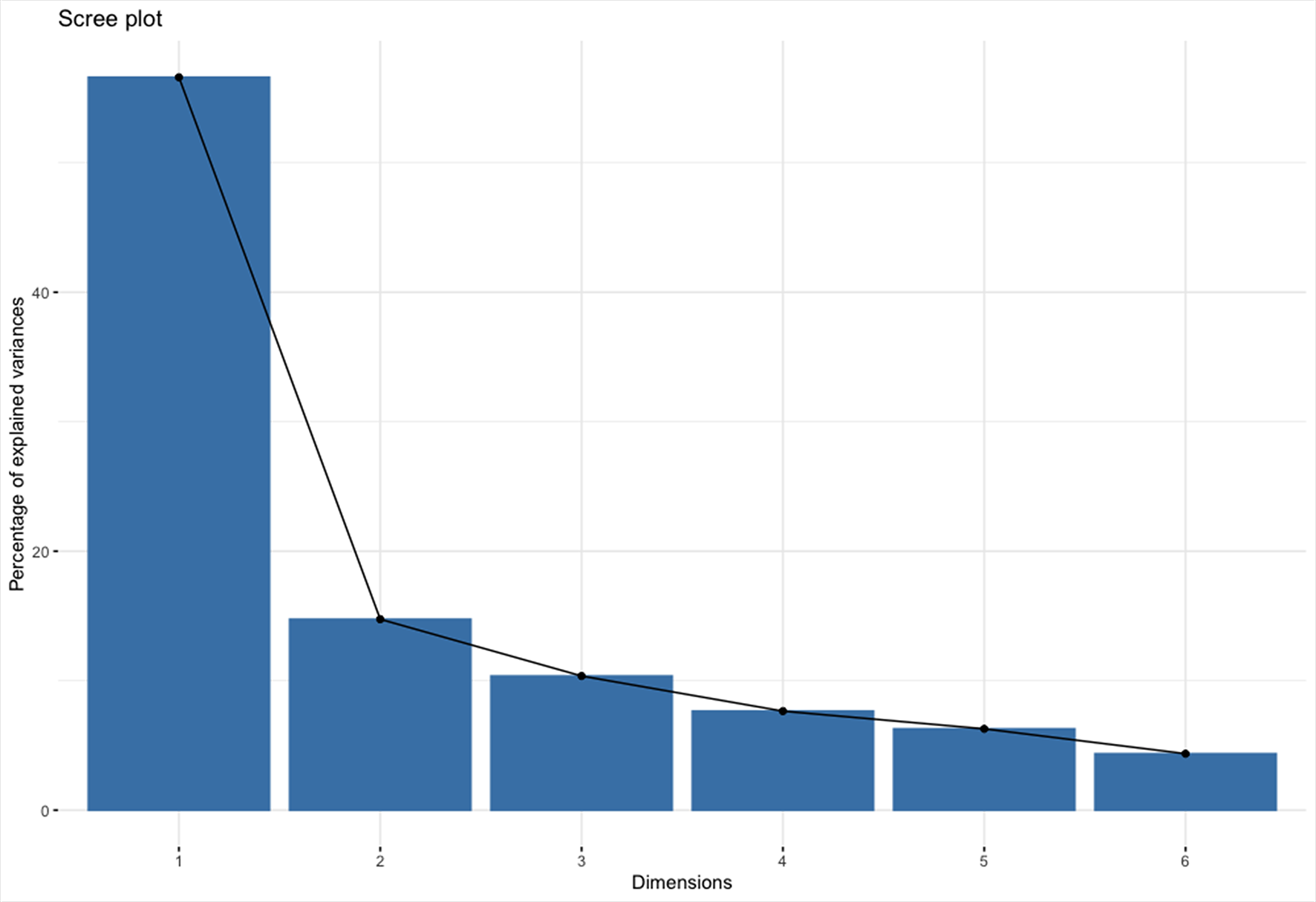

Supplement: Supplementary file 3 [file Image_1.TIFF]

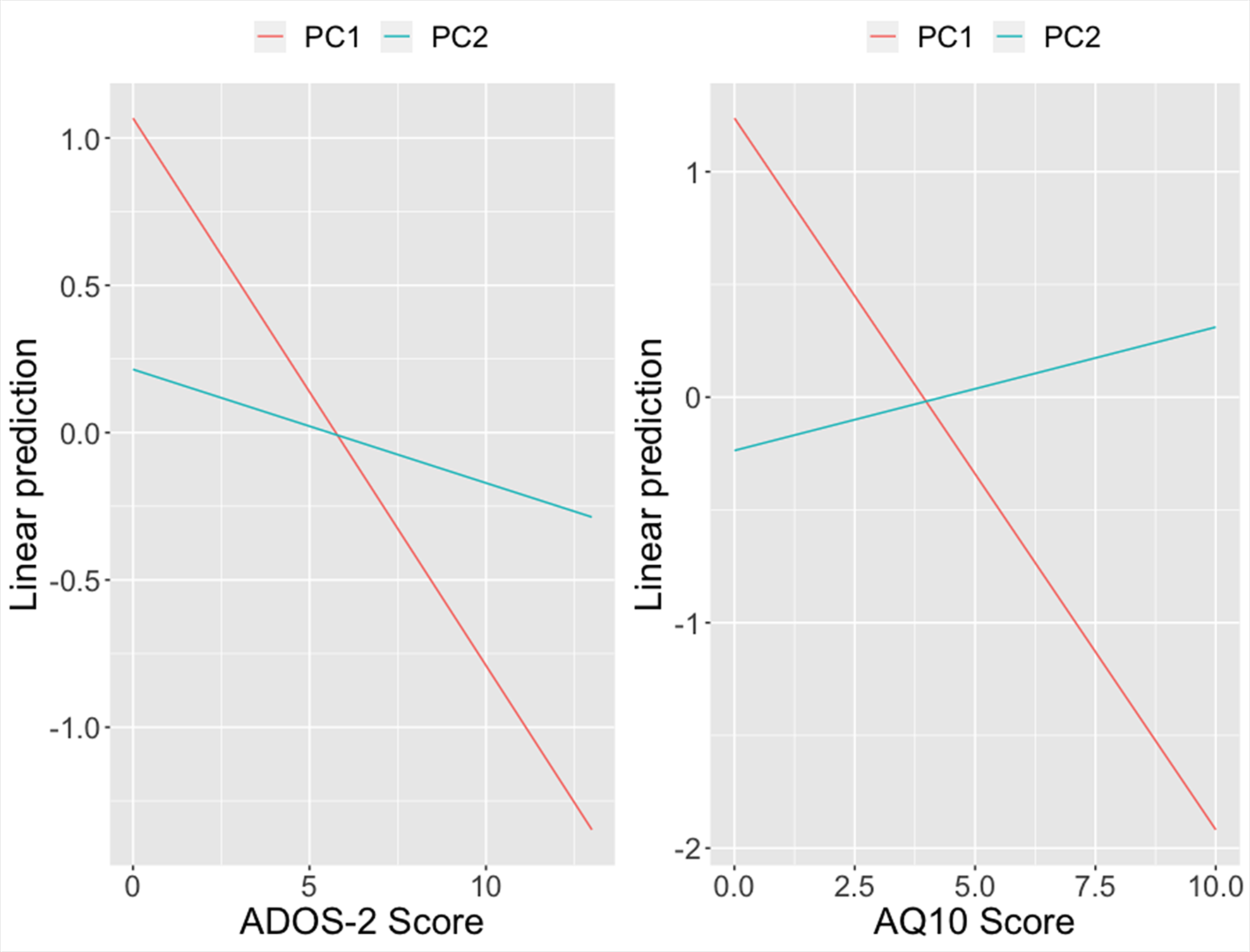

Supplement: Supplementary file 4 [file Image_2.TIFF]
